# Metadherin Mediates Lipopolysaccharide-Induced Migration and Invasion of Breast Cancer Cells

**DOI:** 10.1371/journal.pone.0029363

**Published:** 2011-12-16

**Authors:** Yuhan Zhao, Xiaoli Kong, Xiaoyan Li, Shi Yan, Cunzhong Yuan, Wenwei Hu, Qifeng Yang

**Affiliations:** 1 Department of Breast Surgery, Shandong University School of Medicine, Jinan, Shandong, China; 2 Department of Obstetrics and Gynecology, Qilu Hospital, Shandong University School of Medicine, Jinan, Shandong, China; 3 Cancer Institute of New Jersey, University of Medicine and Dentistry of New Jersey, New Brunswick, New Jersey, United States of America; Sun Yat-sen University Medical School, China

## Abstract

**Background:**

Breast cancer is the most prevalent cancer in women worldwide and metastatic breast cancer has very poor prognosis. Inflammation has been implicated in migration and metastasis of breast cancer, although the exact molecular mechanism remains elusive.

**Principal Findings:**

We show that the pro-inflammatory endotoxin Lipopolysaccharide (LPS) upregulates the expression of Metadherin (MTDH), a recently identified oncogene, in a number of breast cancer lines. Stable knockdown of MTDH by shRNA in human breast MDA-MB-231 cells abolishes LPS-induced cell migration and invasion as determined by several *in vitro* assays. In addition, knockdown of MTDH diminishes Nuclear Factor-kappa B (NF-κB) activation by LPS and inhibited LPS-induced IL-8 and MMP-9 production.

**Conclusions:**

These results strongly suggest that MTDH is a pivotal molecule in inflammation-mediated tumor metastasis. Since NF-κB, IL-8 and MMP-9 play roles in LPS-induced invasion or metastasis, the mechanism of MTDH-promoted invasion and metastasis may be through the activation of NF-κB, IL-8 and MMP-9, also suggesting a role of MTDH in regulating both inflammatory responses and inflammation-associated tumor invasion. These findings indicate that MTDH is involved in inflammation-induced tumor progression, and support that MTDH targeting therapy may hold promising prospects in treating breast cancer.

## Introduction

Breast cancer is the most prevalent cancer in women worldwide and metastatic breast cancer has very poor prognosis. Although breast cancer incidence rate has been decreasing in the past decades due to the early detection, breast cancer remained to be of the top incidence rate and it has the second highest cancer mortality in women [1]. Breast cancer is a heterogeneous disease and is stratified by race, stage, grade, and estrogen (ER)/progesterone (PR) receptor status. Typically, there are two broad categories of genetic changes in the process of tumorigenesis: tumor suppressor genes and oncogenes. Tumor suppressor genes, including BRCA2, inhibit cell division, survival, or other properties of cells. They are often disabled in cancer cells therefore to promote the malignant changes. Oncogenes promote malignancy by expressing at inappropriately high levels, or being altered to have novel properties [2]. Metadherin (MTDH) is a recently identified oncogene [3]. Here we report the role of MTDH in promoting invasion and metastasis in breast cancers.

MTDH, (also known as astrocyte elevated gene-1, AEG-1 and Lyric), is a newly cloned gene, which has aberrantly higher copy numbers at 8q22 in breast cancer patients [4]. MTDH is a 64 kDa single transmembrane protein and located in the cytoplasm, endoplasmic reticulum, perinuclear regions, and nucleolus [5], [6]. The expression of MTDH has been detected in melanoma, glioma, neuroblastoma, and carcinomas of breast, prostate, liver, kidney, colorectum and esophagus [7], [8], [9]. The expression levels of MTDH is positively correlated with tumorigenesis, migration, invasion, angiogenesis, EMT (epithelial mesenchymal transition) and chemoresistance in various cancer types [3], [10], [11], [12], [13].

Current studies have revealed that MTDH could be a prognostic factor in breast cancer: its high expression is associated with poor survival [14]. Statistical analysis showed a significant correlation of MTDH expression with the clinical staging of the patients, tumor classification, node classification, and metastasis classification. Previous studies from our group have also shown a significant correlation between MTDH expression with patients' age, ER status and p53 status that are also poor prognostic features, further supporting the notion that MTDH expression is correlated with poor prognosis and high morbidity in breast cancer patients [15]. Previously, MTDH has been shown to induce the lung metastasis with a “lung-homing domain” selected from lung-homing Balb/c-derived 4T1 mammary tumor cell line phage cDNA library and has been related to tumor angiogenesis with the expression of vascular endothelial growth factor (VEGF) and microvessel density (MVD) [16], [17]. Our group also demonstrated the role of MTDH in promoting metastatic seeding and enhancing chemoresistance [4]. Recently we found that MTDH enhanced EMT which drove the aggressive behavior of the breast cancer and identified novel SNPs of MTDH that are correlated to breast cancer susceptibility [12], [18]. Recently the MTDH/AEG-1-based DNA vaccine was shown to increase chemosensitivity to doxorubicin in inhibiting breast cancer metastasis to the lung[19]. These studies suggested MTDH as a potential candidate of target therapy for cancer, especially for enhancing the efficacy of chemotherapy and reducing metastasis.

About 150 years ago, Virchow first discovered the relationship between chronic inflammation and carcinogenesis [20], which attracted extensive studies. The last decade witnessed increased interest and extensive studies on tumor microenvironment that contributes to neoplastic process, cell proliferation and migration [21]. Among those factors such as the recruitment of microenvironmental cells and the cytokines those cell secreted into the tumor microenvironment, the activation of TLR4 was considered as a two edged sword, which has both the anti-tumor and pro-tumor functions [22]. Lipopolysaccharide (LPS), the major component of the outer membrane of Gram-negative bacteria, also the ligand of TLR4, could up-regulate the expression of MTDH in human promonocytic cell line which takes part in the regulation of the TLR4 signaling pathway, including the activation of Nuclear Factor-kappa B (NF-κB). These observations suggest that MTDH might play an important role in the regulation of innate immunity [11]. NF-κB can promote inflammation-associated tumerigenesis and is related to the MTDH-associated tumor progression and metastasis in cervical cancers [23], [24]. It remains elusive whether MTDH plays a causative role in LPS-induced tumor progression and metastasis. Here we determined the function of MTDH in promoting tumor progression in response to LPS and investigated how LPS regulated MTDH expression in breast cancer cells.

## Results

### LPS induced the upregulation of MTDH in the TLR4-positive breast cancer cells

**Figure 1:**
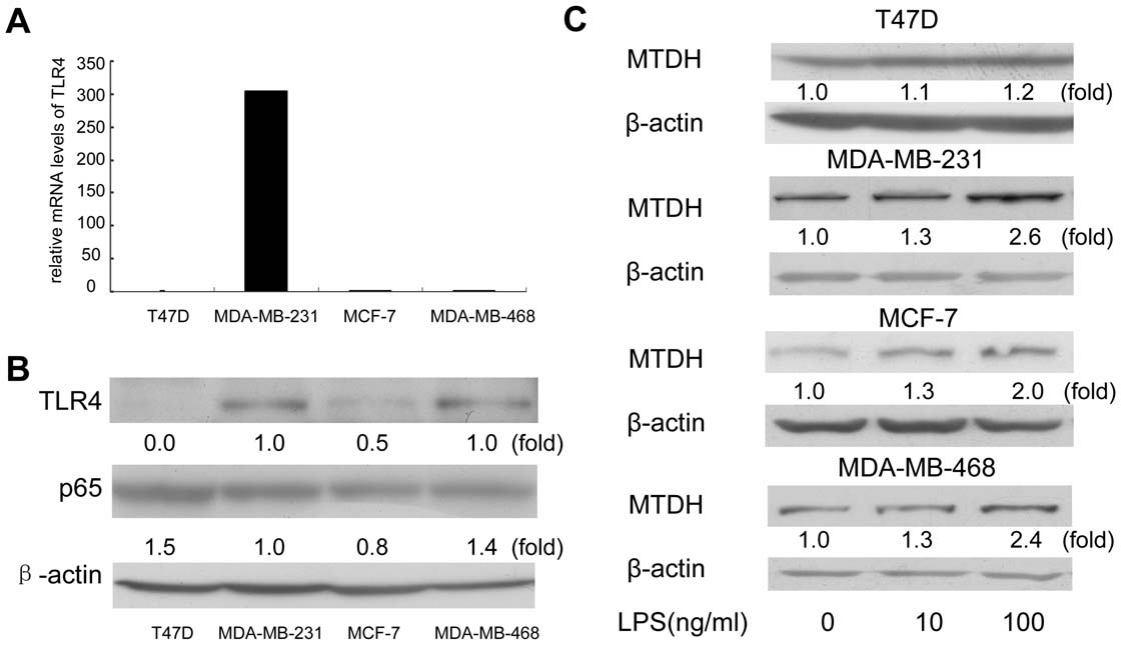
LPS upregulates MTDH expression in TLR4 positive breast cancer cell lines. (**A, B**) The mRNA levels (**A**) and the protein expression levels (**B**) of TLR4 were measured by Real Time RT-PCR and western blots respectively. Total RNA and protein were extracted from the T47D, MDA-MB-231, MCF-7 and MDA-MB-468 cell lines. (**C**) MTDH expressions at protein level in different cell lines were analyzed by western blots with the stimulation of various concentrations of LPS for 1 hour. β-actin was selected to be the endogenous control.

**Figure 2:**
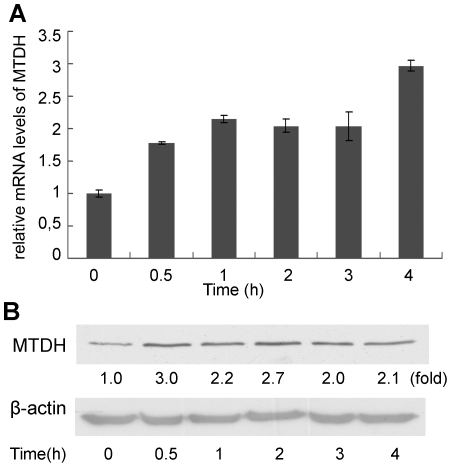
LPS upregulates MTDH expression in a time-dependent manner. (**A, B**) MDA-MB-231 cells were cultured with LPS (100 ng/ml) for various times and the expression of MTDH was examined at both mRNA (**A**) and protein (**B**) levels., β-actin was selected to be the endogenous control.

First, we examined which breast cancer cell lines were TLR4 positive. We evaluated the expression of TLR4 at mRNA level by Real Time RT-PCR. TLR4 expression was detected in three breast cell lines including MDA-MB-231, MCF-7 and MDA-MB-468. TLR4 was undetectable in the cell line T47D, which was used as negative control in our study. The mRNA level of TLR4 was higher in MDA-MB-231 cells than MCF-7 and MDA-MB-468 (Figure 1A). Although the differences of TLR4 protein levels were not so obvious perhaps because of translation control, the expression levels of TLR4 protein in these four cells still showed similar pattern as determined by the Western-blot assays (Figure 1B). Next, we examined the effects of LPS on the expression of MTDH in these cells. As shown in Figure 1C, the expression of MTDH protein changed in a concentration-dependent manner, the peak appeared in the three TLR4 positive cells (MDA-MB-231, MCF-7, and MDA-MB-468) when cells were stimulated with LPS at the concentration of 100 ng/ml. In the TLR4 negative T47D cell line, no significant change of MTDH expression was observed when the cells were treated with LPS. The basal total p65 expression was not significantly different among the four types of cell lines as shown in Figure 1B, suggesting that the different reactions to the LPS stimulation were not due to the lack of basal NF-κB expression. Since MTDH expression level peaked at a concentration of LPS 100 ng/ml, in the following experiments we selected MDA-MB-231 cells as the representative TLR4 positive cells and T47D as TLR4 negative cells treated with 100 ng/ml LPS. We evaluated MTDH expression at mRNA and protein levels at different time points in MDA-MB-231 cells which show clear induction of MTDH by LPS (Figure 2). The results suggested that LPS upregulated the MTDH expression in time-dependent and concentration-dependent manners in TLR4 positive breast cancer cells. There were reports that MTDH was activated over a long period through PI3K/AKT while it was activated within 30 min through the NF-κB signal pathway [11].

### LPS induced up-regulation of MTDH and increased the migration ability of MDA-MB-231 cells

**Figure 3:**
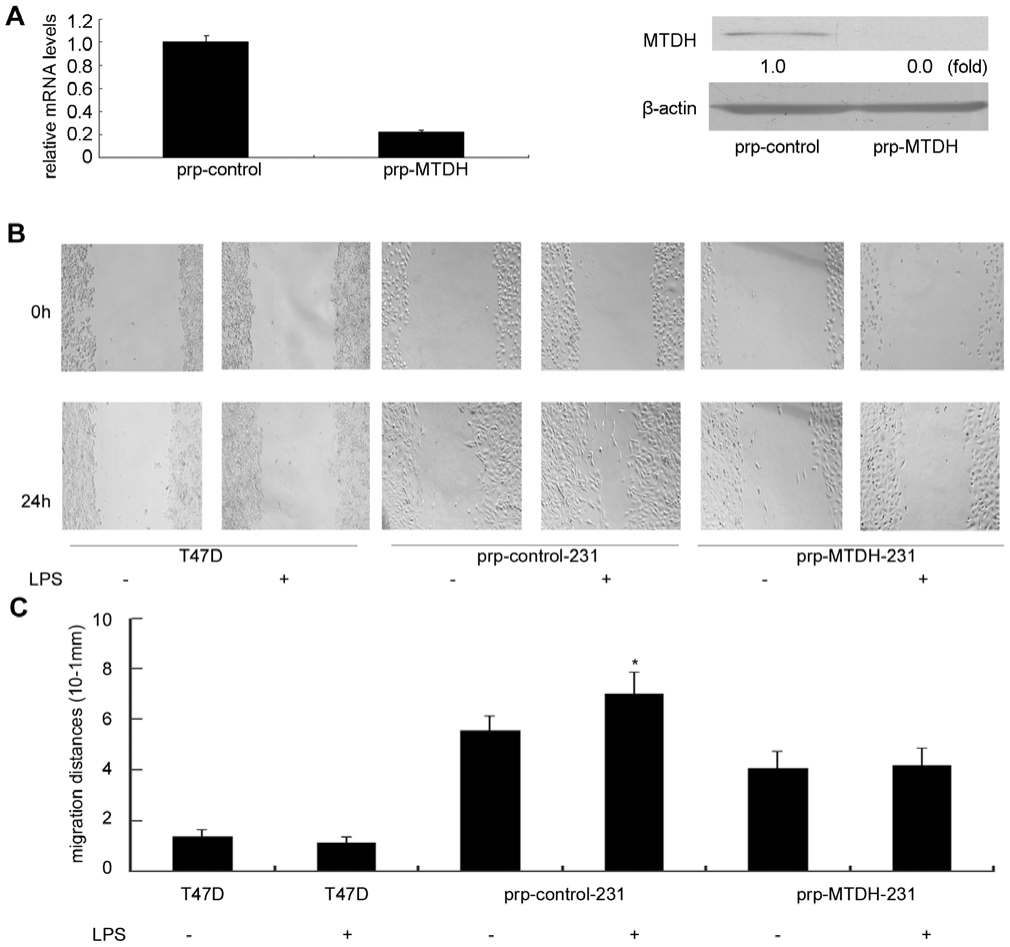
The *in vitro* scratch assays show increased migration ability in LPS-treated MDA-MB-231 cells. (**A**) Relative MTDH mRNA levels and protein levels in MDA-MB-231 cells transfected with prp-control or prp-MTDH vector respectively were measured by Real Time RT-PCR and western blot, respectively. (**B**) Images of un-pretreated or 100 ng/ml LPS-pretreated prp-control and prp-MTDH transfected MDA-MB-231 cells were taken at 0 and 24 hours. Images are shown at the original magnification of ×100. (**C**) Cell motility was quantified by measuring the distance between the migrating cell boundaries. Data were presented as mean±SD, n = 5. Significant difference on wound distance was observed between prp-control-MDA-MB-231 (prp-control) and prp-MTDH-MDA-MB-231 (prp-MTDH) cells, LPS-pretreated and un-pretreated prp-control cells, LPS-pretreated prp-control and prp-MTDH MDA-MB-231 cells. No significant differences was observed between LPS-pretreated and un-pretreated T47D and prp-MTDH-231 cells. T-test was carried out to examine the differences, *:p<0.05 was considered significant compared with the LPS untreated cells.

Since MTDH is considered to be involved in tumor migration and invasion [13], and the LPS induced inflammation response upregulates the MTDH expression in breast cancer cells, we carried out the *in vitro* scratch assays to investigate whether elevated MTDH levels are responsible for the enhanced tumor migration. We knocked down the MTDH expression by shRNA against MTDH. The decrease of MTDH was confirmed at both protein and mRNA levels (Figure 3A). Knockdown of MTDH in MDA-MB-231 cell significantly reduced the migration ability. As shown in Figure 3B, C, MTDH knockdown resulted in a significant decrease in the cell motility after 24 hours of migration with cell migrated (5.56±0.59)×10^−1^ mm (all the starting wound distance was approximately 1 mm) in prp-control cells while in prp-MTDH cells the distance was only (4.06±0.67)×10^−1^ mm. LPS treatment did not change the migration in TLR4 negative T47D cells, however, clearly increased migration in TLR4 positive MDA-MB-231 cells. In the prp-control-231 cells, LPS pretreated cells migrated (7.02±0.87)×10^−1^ mm compared with (5.56±0.59)×10^−1^ mm in un-pretreated cells, the difference was significant (p = 0.02). However, this kind of change was not observed in the MTDH knockdown (prp-MTDH-231) cells when the data showed cell motilities were (4.18±0.68)×10^−1^ mm and (4.06±0.67)×10^−1^ mm in LPS pretreated and un-pretreated cells (p = 0.39). This change demonstrated that elevated MTDH expression was required for LPS-induced breast cancer cell migration.

### LPS increased the invasive ability of MDA-MB-231 cells

**Figure 4:**
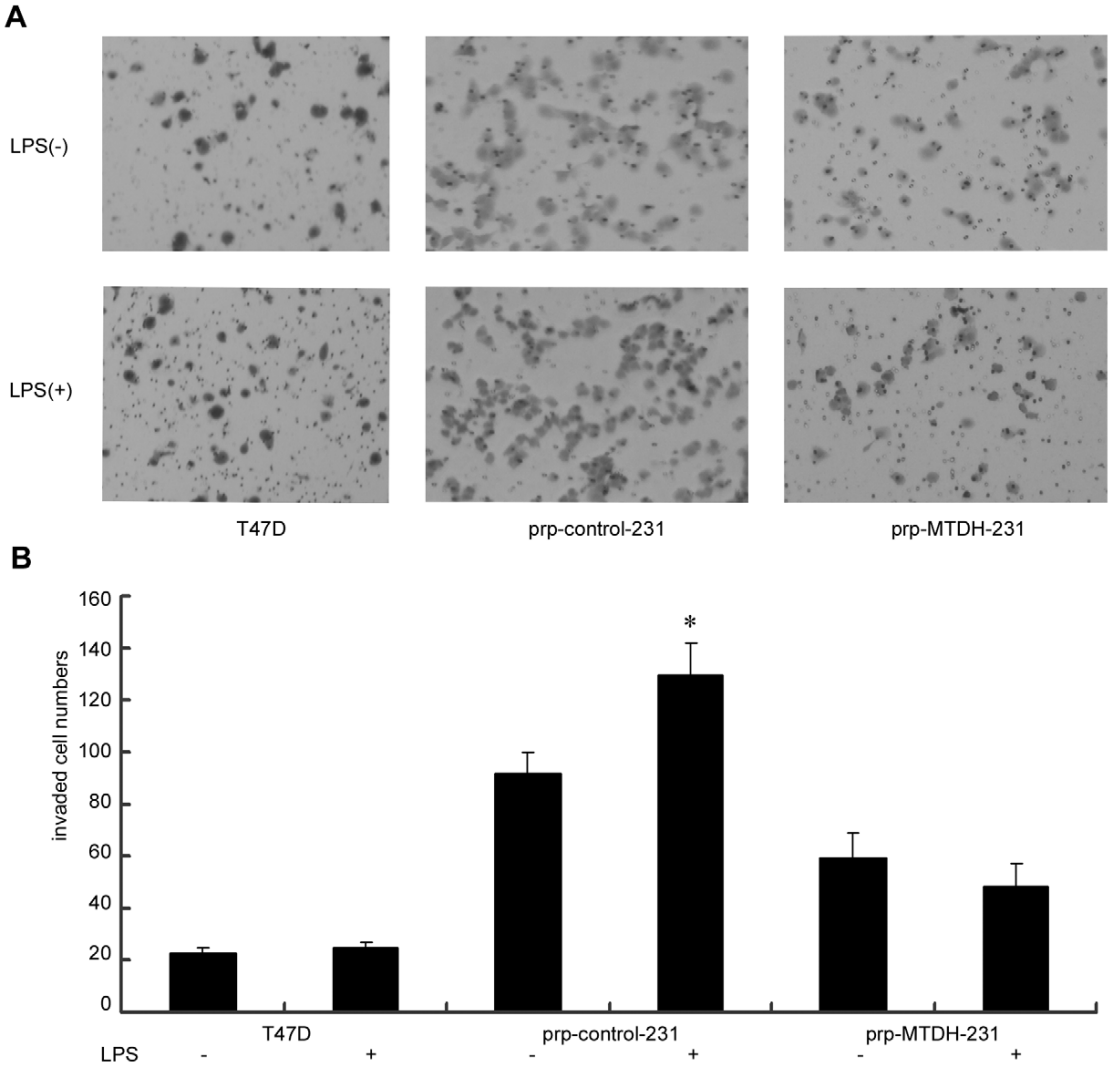
Knockdown of MTDH abolishes LPS-induced breast cancer cell invasion. (**A**) Images were taken from invasion assays using transwell system with or without the stimulation of LPS (100 ng/ml) for 18 hours in T47D, prp-control and prp-MTDH MDA-MB-231 cells. Images are shown at the original magnification of ×100. (**B**) The number of successfully invaded cells. The cell numbers were counted at five representative fields. Data was presented as mean±SD. The invaded cell number difference between T47D cells without LPS stimulation (22.6±2.1) and with LPS stimulation (25.2±1.9) was not significant (p = 0.074). The invaded cell number difference between prp-control MDA-MB-231 cells (92.2±8.6) and LPS-treated prp-control MDA-MB-231 cells (129.8±12.6) was significant; so was that between prp-control and prp-MTDH MDA-MB-231 cells with or without LPS stimulation, *:p<0.05 was considered significant compared with the LPS untreated cells.

Since MTDH mediates LPS-induced cell migration, we went on to examine the effect of LPS-induced MTDH on invasion in MDA-MB-231 cells. First we examined whether LPS affected the proliferation ability of the MDA-MB-231 cells. MDA-MB-231 cells were cultured with various concentrations of LPS (0.01, 0.1, 1.0, 10 ug/ml) for 24, 48, 72, 96 hours and the proliferation of cells were measured. LPS did not have obvious effect on the proliferation of MDA-MB-231 cells (data not shown). The invasion ability of the cells was significantly increased upon LPS treatment (100 ng/ml) for 18 hours in TLR4 positive cells. In TLR4 negative T47D cells, there was no change in invasion ability. Interestingly, the effect of LPS on promoting invasion was completely abolished by MTDH knockdown (Figure 4). These results strongly suggest that LPS increased the invasive ability of MDA-MB-231 cells through the up-regulation of MTDH expression.

### LPS induced MTDH expression regulated the TLR4 signaling pathways

**Figure 5:**
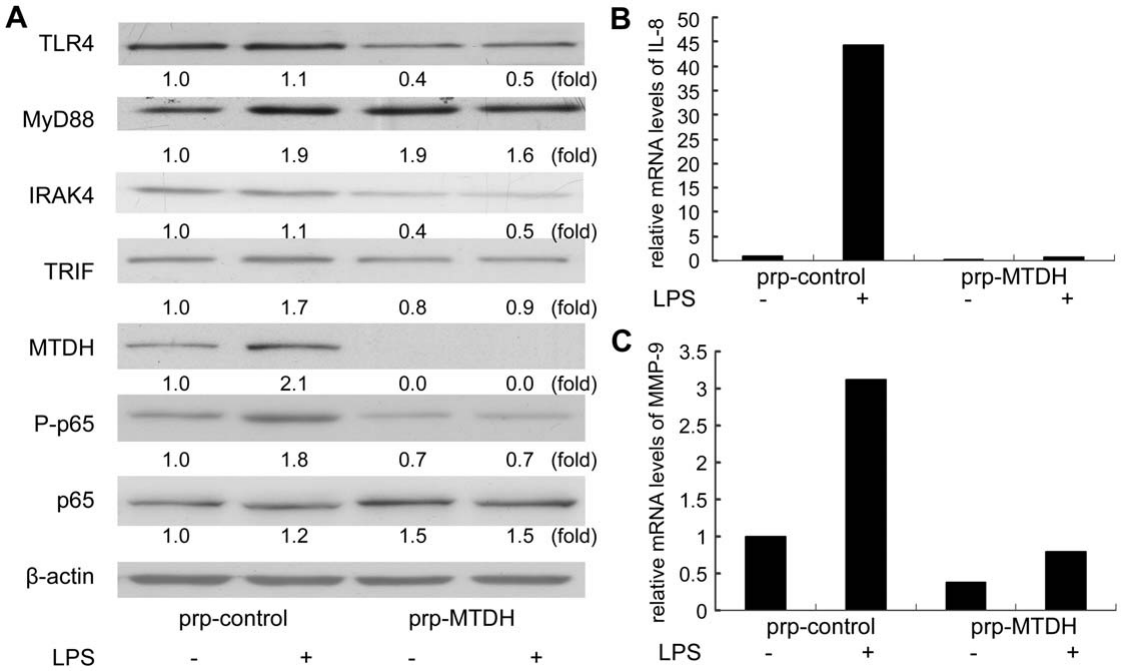
MTDH regulates LPS-induced NF-κB activation and cytokine production. (**A**) Protein expression levels were determined by Western-blot assays. Protein extracts were prepared from the prp-control and prp-MTDH MDA-MB-231 cells treated without or with LPS (100 ng/ml) for 1 hour. (**B, C**) The mRNA levels of IL-8 (**B**) and MMP-9 (**C**) were measured by Real Time RT-PCR. Total RNA was extracted from cells in with or without of LPS (100 ng/ml) for 24 hours. β-actin was used as an endogenous control. Each experiment was carried out three times independently.

The TLR4 signaling pathway includes MyD88 (myeloid differentiation primary response gene 88)-dependent and MyD88-independent pathways [25]. It has been reported that the early-phase NF-κB activation by LPS was dependent on MyD88, whereas late-phase NF-κB activation was dependent on TRIF (TIR domain-containing adaptor inducing IFN-β,mediating MyD88-independent signaling), and both phases of NF-κB activation were required for cytokine production [26]. We evaluated the MYD88, IRAK4 (IL-1 receptor-associated kinase 4) expressions which were in the MYD88-dependent pathway and TRIF which was involved in MYD88-independent pathway by Western-blot assays. The expression levels of TLR4, IRAK4, TRIF and P-p65 were decreased in MTDH knockdown cells (Figure 5A). As shown in Figure 5A, all the protein mentioned above in the TLR4 signaling pathway were upregulated when stimulated by LPS, and in the MTDH knockdown cells the TLR4 and TRIF were slightly upregulated meanwhile MyD88, IRAK4 expression changes were not obvious. Those results implied that LPS activated MTDH in the TLR4 signaling pathway and the depletion of MTDH turned out to further impede this pathway.

The NF-κB family (also known as the Rel/NF-κB family) of transcription factors consists of five members: p50, p52, RelA (also known as p65), RelB and c-Rel. With the activation of NF-κB, the p65 was phosphorylated; the p50-p65 NF-κB translocated into the nucleus, which in turn bound to the specific sequence and augmented related downstream gene transcription. LPS treatment in prp-control cells increased the P-p65 level (Figure 5A), suggesting that NF-κB was activated by LPS-induced upregulation of MTDH, which is consistent with the previous study that MTDH activates the NF-κB pathway [27]. In the MTDH knockdown cells, the P-p65 was barely detectable with or without the stimulation of LPS, providing evidence that MTDH mediates the activation of NF-κB by LPS.

### Cytokines production changes detected with the stimulation of LPS

IL-8 and MMP-9 are the NF-κB downstream genes and their expressions were examined by Real Time RT-PCR. When MTDH was knocked down, the expressions of both IL-8 and MMP-9 decreased to 0.16-fold and 0.38-fold of those expressed in prp-control cells (Figure 5B, C). With the stimulation of LPS, both IL-8 and MMP-9 expression in prp-control cells increased corresponding to the upregulated expression of MTDH. LPS treatment increased the expression of IL-8 by ∼44-fold and MMP-9 by ∼3-fold in prp-control cells; in prp-MTDH cells, the induction of IL-8 and MMP-9 by LPS was ∼4-fold and ∼2-fold, respectively. These results indicated that IL-8 and MMP-9 are the downstream genes of MTDH.

## Discussion

Cancer-related inflammation is an important hallmark of cancer [28], [29]. Inflammation induces the genetic instability in the tumor microenvironment [30]. Two pathways have been identified linking inflammation and cancer: the extrinsic ones and intrinsic ones [31]. In the extrinsic pathway, inflammatory environment promotes the malignancy of cancer. For example, the Helicobacter pylori infection increases the risk of gastric cancer and hepatitis virus infection is found in majority of liver cancers. Here, our study provided the evidence that LPS could upregulate the expression of MTDH which increases the invasiveness of breast cancer. In the intrinsic pathway, genetic changes lead to the expression of inflammation-related programs that guide the construction of an inflammatory microenvironment. The production of cytokines such as IL-8 and MMP-9 was increased, which could further help inducing angiogenesis and modifying the extracellular matrix in the tumor microenvironment. In our present study, we found that MTDH is a molecule linking inflammation and cancer.

Toll-like receptors (TLRs) are a family of pattern recognition receptors that are best-known for their role in initiation of the innate and adaptive immune responses. However, recent evidences showed that functional TLRs are also expressed in a wide variety of tumors suggesting that TLRs may play important roles in tumor biology. An earlier report revealed that TLR4 was expressed on many tumor cell surfaces and associated with the risk of prostate cancer, nasopharyngeal carcinoma and gastric carcinoma [22]. LPS, the ligand of TLR4, was reported to increase the lung metastasis in 4T1 mouse mammary carcinoma cells [32]. Furthermore, a recent study showed that samples of breast carcinomas with recurrence exhibit a significant increase in the mRNA levels of TLR4 and that TLR4 expression is significantly associated with a great rate of distant metastasis [33]. Also the activation of NF-κB, which is the downstream gene of TLR4, contributed to mammary epithelial tumor progression *in vivo.* Inhibition of NF-κB led to the decreased tumor burden and decreased numbers of lung metastases [34]. All these results provide a possibility that TLR4- NF-κB pathway is a connection between inflammation, immunity and cancer.

In our study, TLR4 expressed in breast cancer cell line MDA-MB-231, MCF-7 and MDA-MB-468. We demonstrated the activation of NF-κB with the stimulation of LPS in accordance with the overexpression of MTDH. Previously, MTDH was reported as a LPS-responded gene in proinflammatory cells which suggested that MDTH may play an important role in the regulation of innate immunity [11]. Moreover, MDTH is also involved in tumor progression, migration and invasion as a NF-κB dependent gene in Hela cells [24], [27]. It functioned as a bridging molecule between NF-κB, CBP (cyclic AMP-responsive element binding protein-binding protein) and the basal transcription machinery. The present study demonstrated for the first time that LPS could increase the MTDH expression via activating the TLR4-NF-κB signaling pathway in human breast cancer cells, we hypothesized that there might be a positive feedback loop that LPS stimulation initiated a signaling cascade leading to upregulated expression of MTDH, which subsequently activated NF-κB, resulting in a second phase of TLR4 activation or TRIF activation, reinforcing and amplifying LPS-induced signaling. Our results provided evidence that MTDH correlated the inflammation, innate immunity and carcinogenesis together.

It has been reported that the LPS-induced metastatic growth was associated with increased angiogenesis vascular permeability and tumor cell invasion [35]. In this study we have shown the increased secretion of IL-8 and MMP-9 by LPS treatment. IL-8 is believed to function as a chemoattractant and a potent angiogenic factor. Furthermore, IL-8 can promote cell invasion and migration, and the capacity of IL-8 to induce tumor-associated macrophages to secrete additional growth factors will further increase the rate of cell proliferation and cancer cell invasion at the tumor site[36]. MMP-9, a 92 kDa type IV collagenase, 92 kDa gelatinase or gelatinase B (GELB), is a key effecter of ECM remodeling. MMP-9 is activated to degrade type IV and V collagens and promote the process of metastasis. In the present study, LPS upregulated the MTDH expression and further activated the NF-κB pathway, promoting the secretion of the downstream genes such as IL-8 and MMP-9 in prp-control-231 cells. With the knockdown of MTDH, the induction of the mRNA expression of IL-8 and MMP-9 decreased profoundly, and LPS treatment did not increase their secretion. This indicated that MTDH increased the metastatic ability of breast cancer via the secretion of these cytokines. Besides, there was study put forward the hypothesis that signaling through TLR4 is strongly inhibited by intact extracellular matrix and that inhibition is abrogated and endogenous agonist(s) are liberated when the matrix is degraded [37]. It is possible that with upregulation of MTDH induced by LPS, the secretion of cytokines such as MMPs increased greatly and could degrade the extracellular matrix which then further activated the TLR4 and enhanced the invasive ability of cancer cells.

In summary, induction of MTDH by LPS could increase the migration and invasiveness in the TLR4 positive breast cancer cells. To date, we are the first to show the role of MTDH in the TLR4 signaling pathway in breast cancer cell line, MDA-MB-231 cells. The results provide evidence that MTDH might be a pivotal molecule not only in the progression of cancers but also in relation of cancer, innate immunity and inflammation. However, the immune evasion possibly mediated by TLR4- NF-κB-MTDH pathway should be further studied. The relationship between MTDH and TLR4 signaling pathway might lead to the development of TLR4 and MTDH as novel therapeutic targets for breast cancer.

## Materials and Methods

### Materials

LPS isolated from Escherichia coli (0111:b4) was purchased from Sigma Chemical Co. (St. Louis, MO). The rabbit polyclonal antibody to TLR4, clone H-80, was purchased from Santa Cruz Biotechnology, Inc. (Santa Cruz, CA). Primary antibodies against MyD88, TRIF, IRAK4, NF-κB p65 and phospho-NF-κB (P-p65) were purchased from Cell Signaling Technology (Beverly, MA, USA). Dulbecco's Modified Eagle's Medium (DMEM), RPMI Medium 1640 were purchased from Gibco-BRL (Rockville, IN, USA). Fetal bovine serum (FBS) was supplied by Haoyang Biological Manufacturer Co., Ltd (Tianjin, China).

### Cell culture

Breast cancer cell lines T47D, MDA-MB-231, MCF-7 and MDA-MB-468 were obtained from American Type Culture Collection (ATCC). Breast cancer cells T47D were cultured in RPMI-1640 supplemented with 10% FBS and 0.2 U/ml insulin while MDA-MB-231, MCF-7 and MDA-MB-468 were routinely cultured in DMEM supplemented with 10% FBS and 100 units of penicillin-streptomycin at 37°C with 5% CO2 in a humidified incubator. For LPS treatment, LPS was added in the culture medium for the indicated times and concentrations.

### Western blot analysis

Cells were washed three times with cold phosphate-buffer saline (PBS) and lysed on ice in RIPA (1×PBS, 1% NP40, 0.1% sodium dodecyl sulfate (SDS), 5 mM EDTA, 0.5% sodium deoxycholate and 1 mM sodium orthovanadate) with protease inhibitors. The concentrations of protein were determined by BCA methods. Eighty micrograms of protein were separated by 10% SDS-PAGE and electro-blotted to a PVDF membrane using a semi-dry blotting apparatus (Bio-Rad, Hercules, CA, USA). After blocked in 5% non-fat milk, the membranes were incubated overnight at 4°C with the primary antibodies. Then membranes were incubated in the secondary antibodies for 2 hours at room temperature with slightly shock. The bands are visualized using a Pro-lighting HRP agent. β-actin was used as a loading control.

### Real-Time RT-PCR

Total RNA was extracted with TRIZOL reagents according to the manufacturer's protocol (invitrogen, Carlsbad, CA, USA). The cDNA was synthesized from 800 ng of total RNA by PrimerScript RT Reagent Kit (TaKaRa BIO Inc, Otsu, Shiga, Japan). The real-time RT-PCR was carried out by using a SYBR green PCR mix in Applied Biosystems StepOne and StepOnePlus Real-Time PCR Systems. The gene expression values were calculated by normalizing with endogenous control β-actin with DataAssist software. Each experiment was repeated three times to confirm the conclusions.

### Plasmids construction and Cell transfection

The plasmids used in the experiment were described previously (Hu, Chong et al. 2009). Briefly, two different short-hairpins RNA (shRNA) were constructed to knockdown the expression of MTDH in MDA-MB-231 cell line, generating the psuper-retro-puro-MTDH (prp-MTDH) cells, psuper-retro-puro vector was used as control (prp-control). Cells were seeded in six-well plates 24 hours before the transfection and were transfected with psuper-retro-puro-MTDH or psuper-retro-puro vectors by Lipofectamine 2000 transfection reagent (Invitrogen, USA) according to the manufacturer's instructions. Cells were selected for 2 weeks in 0.5 ug/ml puromycin and maintained in 0.2 ug/ml puromycin. The expression levels of MTDH in surviving colonies were evaluated by western blot and RT-PCR.

### *In vitro* scratch assays

The cells were seeded in 24-well plates at a density 1×10^5^ cells/well. After incubation overnight, cells were stimulated with LPS (100 ng/ml) for 4 hours, and then scraped by a p20 pipette tips to create a straight-line cell-free scratch. Each well was washed with PBS three times to remove the remaining unattached cells and debris. The scratch area were marked and photographed in every 12 hours. The distances were measured by the software imageJ and cell motility was quantified by measuring the distance between the migrating cell boundaries [38]. Data were analyzed statistically.

### Invasion assays

The transwell system (24 wells, 8 um pore size, BD Biosciences, Heidelberg, Germany) coated with 2 mg/ml basement membrane Matrigel (BD Biosciences) were used for the in vitro invasion assays. 1×10^5^ cells were suspended in serum-free DMEM in the upper chamber of each well. The lower well of each chamber was filled with 750 ul DMEM supplemented with 20% FBS. LPS (100 ng/ml) was added to both the upper and the lower chambers. After 18 hours of LPS treatment, filters were fixed with methanol and stained with 0.1% crystal violet. Then the number of cells in at least five randomly selected microscope fields were counted and analyzed statistically.

### Statistical evaluation

Statistical differences were analyzed by Student's t-test, p<0.05 was considered significant. Independent repeated experiments were carried out at least three times.

## References

[1] Jemal A, Siegel R, Xu J, Ward E (2010). Cancer statistics, 2010.. CA Cancer J Clin.

[2] Knudson AG (2001). Two genetic hits (more or less) to cancer.. Nat Rev Cancer.

[3] Emdad L, Lee SG, Su ZZ, Jeon HY, Boukerche H (2009). Astrocyte elevated gene-1 (AEG-1) functions as an oncogene and regulates angiogenesis.. Proc Natl Acad Sci U S A.

[4] Hu G, Chong RA, Yang Q, Wei Y, Blanco MA (2009). MTDH activation by 8q22 genomic gain promotes chemoresistance and metastasis of poor-prognosis breast cancer.. Cancer Cell.

[5] Sutherland HG, Lam YW, Briers S, Lamond AI, Bickmore WA (2004). 3D3/lyric: a novel transmembrane protein of the endoplasmic reticulum and nuclear envelope, which is also present in the nucleolus.. Exp Cell Res.

[6] Kang DC, Su ZZ, Sarkar D, Emdad L, Volsky DJ (2005). Cloning and characterization of HIV-1-inducible astrocyte elevated gene-1, AEG-1.. Gene.

[7] Kikuno N, Shiina H, Urakami S, Kawamoto K, Hirata H (2007). Knockdown of astrocyte-elevated gene-1 inhibits prostate cancer progression through upregulation of FOXO3a activity.. Oncogene.

[8] Chen W, Ke Z, Shi H, Yang S, Wang L (2010). Overexpression of AEG-1 in renal cell carcinoma and its correlation with tumor nuclear grade and progression.. Neoplasma.

[9] Song H, Li C, Li R, Geng J (2010). Prognostic significance of AEG-1 expression in colorectal carcinoma.. Int J Colorectal Dis.

[10] Emdad L, Sarkar D, Su ZZ, Lee SG, Kang DC (2007). Astrocyte elevated gene-1: recent insights into a novel gene involved in tumor progression, metastasis and neurodegeneration.. Pharmacol Ther.

[11] Khuda II, Koide N, Noman AS, Dagvadorj J, Tumurkhuu G (2009). Astrocyte elevated gene-1 (AEG-1) is induced by lipopolysaccharide as toll-like receptor 4 (TLR4) ligand and regulates TLR4 signalling.. Immunology.

[12] Zhang B, Liu XX, He JR, Zhou CX, Guo M (2011). Pathologically decreased miR-26a antagonizes apoptosis and facilitates carcinogenesis by targeting MTDH and EZH2 in breast cancer.. Carcinogenesis.

[13] Hu G, Wei Y, Kang Y (2009). The multifaceted role of MTDH/AEG-1 in cancer progression.. Clin Cancer Res.

[14] Li J, Zhang N, Song LB, Liao WT, Jiang LL (2008). Astrocyte elevated gene-1 is a novel prognostic marker for breast cancer progression and overall patient survival.. Clin Cancer Res.

[15] Su P, Zhang Q, Yang Q (2010). Immunohistochemical analysis of Metadherin in proliferative and cancerous breast tissue.. Diagn Pathol.

[16] Brown DM, Ruoslahti E (2004). Metadherin, a cell surface protein in breast tumors that mediates lung metastasis.. Cancer Cell.

[17] Li C, Li R, Song H, Wang D, Feng T (2011). Significance of AEG-1 expression in correlation with VEGF, microvessel density and clinicopathological characteristics in triple-negative breast cancer.. J Surg Oncol.

[18] Liu X, Zhang N, Li X, Moran MS, Yuan C (2011). Identification of novel variants of metadherin in breast cancer.. PLoS One.

[19] Qian BJ, Yan F, Li N, Liu QL, Lin YH (2011). MTDH/AEG-1-based DNA vaccine suppresses lung metastasis and enhances chemosensitivity to doxorubicin in breast cancer.. Cancer Immunol Immunother.

[20] Balkwill F, Mantovani A (2001). Inflammation and cancer: back to Virchow?. Lancet.

[21] Coussens LM, Werb Z (2002). Inflammation and cancer.. Nature.

[22] Huang B, Zhao J, Unkeless JC, Feng ZH, Xiong H (2008). TLR signaling by tumor and immune cells: a double-edged sword.. Oncogene.

[23] Pikarsky E, Porat RM, Stein I, Abramovitch R, Amit S (2004). NF-kappaB functions as a tumour promoter in inflammation-associated cancer.. Nature.

[24] Lee SG, Su ZZ, Emdad L, Sarkar D, Fisher PB (2006). Astrocyte elevated gene-1 (AEG-1) is a target gene of oncogenic Ha-ras requiring phosphatidylinositol 3-kinase and c-Myc.. Proc Natl Acad Sci U S A.

[25] Lu YC, Yeh WC, Ohashi PS (2008). LPS/TLR4 signal transduction pathway.. Cytokine.

[26] Inoue J, Gohda J, Akiyama T, Semba K (2007). NF-kappaB activation in development and progression of cancer.. Cancer Sci.

[27] Sarkar D, Park ES, Emdad L, Lee SG, Su ZZ (2008). Molecular basis of nuclear factor-kappaB activation by astrocyte elevated gene-1.. Cancer Res.

[28] Hanahan D, Weinberg Robert A (2011). Hallmarks of Cancer: The Next Generation.. Cell.

[29] Hanahan D, Weinberg RA (2000). The hallmarks of cancer.. Cell.

[30] Colotta F, Allavena P, Sica A, Garlanda C, Mantovani A (2009). Cancer-related inflammation, the seventh hallmark of cancer: links to genetic instability.. Carcinogenesis.

[31] Mantovani A, Allavena P, Sica A, Balkwill F (2008). Cancer-related inflammation.. Nature.

[32] Pidgeon GP, Harmey JH, Kay E, Da Costa M, Redmond HP (1999). The role of endotoxin/lipopolysaccharide in surgically induced tumour growth in a murine model of metastatic disease.. Br J Cancer.

[33] Gonzalez-Reyes S, Marin L, Gonzalez L, Gonzalez LO, del Casar JM (2010). Study of TLR3, TLR4 and TLR9 in breast carcinomas and their association with metastasis.. BMC Cancer.

[34] Connelly L, Barham W, Onishko HM, Sherrill T, Chodosh LA (2011). Inhibition of NF-kappa B activity in mammary epithelium increases tumor latency and decreases tumor burden.. Oncogene.

[35] Harmey JH, Bucana CD, Lu W, Byrne AM, McDonnell S (2002). Lipopolysaccharide-induced metastatic growth is associated with increased angiogenesis, vascular permeability and tumor cell invasion.. Int J Cancer.

[36] Waugh DJJ, Wilson C (2008). The Interleukin-8 Pathway in Cancer.. Clinical Cancer Research.

[37] Brunn GJ, Bungum MK, Johnson GB, Platt JL (2005). Conditional signaling by Toll-like receptor 4.. FASEB J.

[38] Rahim S, Beauchamp EM, Kong Y, Brown ML, Toretsky JA (2011). YK-4-279 Inhibits ERG and ETV1 Mediated Prostate Cancer Cell Invasion.. PLoS One.

